# Supplementary material for: Tead1a Initiates Transcriptional Priming Through the TEAD1a/YAP‐Notch1‐Spi1/Cebpα Axis to Promote Neutrophil Fate
Source: Adv Sci (Weinh). 2025 Aug 28;12(41):e05441. doi: 10.1002/advs.202505441 (PMC12591127; doi:10.1002/advs.202505441)
Supplement: Supplementary file 4 — Supplementary Dataset [file ADVS-12-e05441-s004.zip › 3-1.zip!/3-1/02-1.cluster/cluster0/Enrich/ego_BP.pdf]

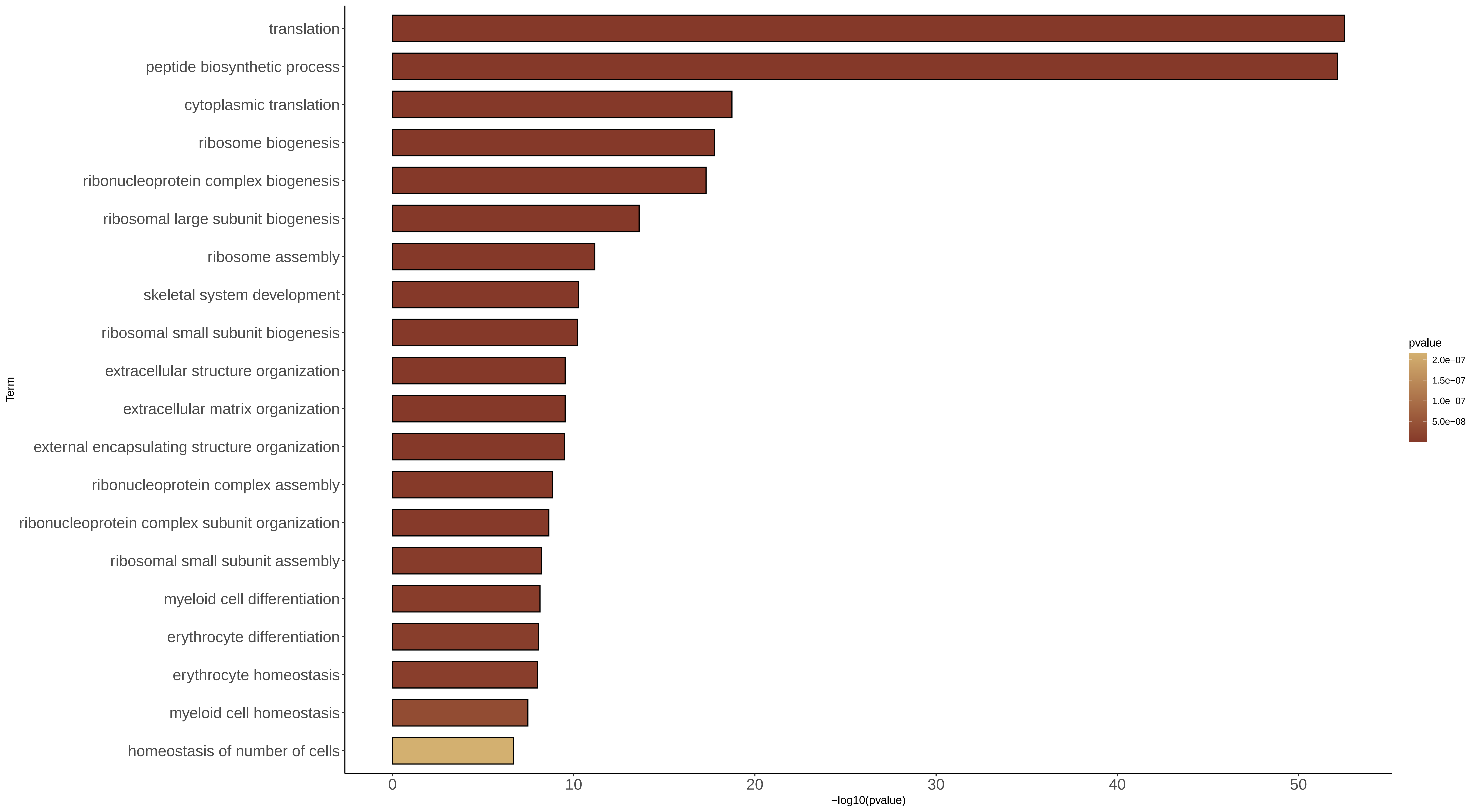
translation
peptide biosynthetic process
cytoplasmic translation
ribosome biogenesis
ribonucleoprotein complex biogenesis
ribosomal large subunit biogenesis
ribosome assembly
skeletal system development
ribosomal small subunit biogenesis
extracellular structure organization
extracellular matrix organization
external encapsulating structure organization
ribonucleoprotein complex assembly
ribonucleoprotein complex subunit organization
ribosomal small subunit assembly
myeloid cell differentiation
erythrocyte differentiation
erythrocyte homeostasis
myeloid cell homeostasis
homeostasis of number of cells
Term
0
10
20
30
40
50
−log10(pvalue)
pvalue
2.0e−07
1.5e−07
1.0e−07
5.0e−08
